# Genomic and functional divergence of oxalate metabolism pathways in bacteria from contrasting ecosystems

**DOI:** 10.1099/mgen.0.001587

**Published:** 2026-02-20

**Authors:** Arun N. Prasanna, Sabiha Parween, Katja Froehlich, Tanja Schmidt, Kirti Shekhawat, Durga D. Prabhu, Maged M. Saad, Heribert Hirt

**Affiliations:** 1 DARWIN21, Biological and Environmental Science and Engineering Division, King Abdullah University of Science and Technology (KAUST), 23955-6900 Thuwal, Saudi Arabia; 2 Max Perutz Laboratories, University of Vienna, Vienna, Austria

**Keywords:** oxalotrophy, metabolic pathways, extreme environments, genome assembly, oxalic acid

## Abstract

Oxalotrophy refers to the ability of bacteria to utilize oxalate as a carbon and energy source. This is a critical process, with significant implications for the global carbon cycle. Oxalate-degrading bacteria play a key role in carbon sequestration through the oxalate–carbonate pathway, contributing to stable inorganic carbon pools. In this study, we identified and catalogued 19 enzymes and a transporter associated with various facets of oxalate metabolism to characterize the oxalotrophic potential of bacteria. Within this group, sets of enzymes were grouped into two functional categories in the context of carbon sequestration: a biomineralization toolkit for converting oxalate to inorganic carbon and an assimilation toolkit for incorporating oxalate into metabolic pathways such as amino acid biosynthesis and energy production. Using bioinformatic approaches, we analysed a collection of 536 bacterial genomes from desert and dryland strains spanning 81 genera to identify oxalotrophs. To validate our findings, we tested several bacterial strains for growth on media supplemented with exogenous oxalate. Notably, while multiple bacterial strains grew on oxalate media, two *Pseudomonas* species, namely JZ043 and JZ097, failed to grow despite genomic predictions suggesting otherwise. Further investigation of these strains revealed several non-conservative amino acid substitutions in the glyoxylate carboligase enzyme (EC 4.1.1.47), a key player in oxalate metabolism, suggesting a potential link between these mutations and their inability to metabolize oxalate. Our findings highlight the significance of our approach for identifying oxalotrophic bacteria and offer valuable insights into the molecular basis of oxalate metabolism.

## Graphical Abstract

Oxalotrophic bacteria from arid soils utilize oxalate via biomineralization (OCP) or assimilation pathways, influenced by key enzymes, shaping ecological roles and oxalate metabolism phenotypes in diverse genera.

**Impact Statement**

Our work provides a comprehensive mapping of 19 oxalate-metabolizing enzymes and a transporter across 536 bacterial genomes from dryland ecosystems and mangroves, revealing diverse biomineralization and assimilation toolkits that drive carbon sequestration. By linking non-conservative mutations in glyoxylate carboligase to the failure of predicted oxalotrophs to grow on oxalate, we demonstrate the power of integrated bioinformatics and functional validation in accurately identifying oxalotrophic bacteria. These insights refine our understanding of microbial contributions to the oxalate–carbonate pathway and inform strategies for harnessing oxalotrophs in carbon capture and soil health applications.

## Data Availability

All the data used in this study are publicly available in the National Center for Biotechnology Information (NCBI) Genomes, Sequence Read Archive (SRA), European Nucleotide Archive (ENA), and in-house repository.

## Data Summary

The dataset contains genomes and raw sequence reads deposited in the NCBI Genomes, SRA, and ENA repositories. Besides, all the assemblies used in this study are deposited in the DARWIN21 repository, our in-house database (https://www.genomedatabase.org/) [1]. The accessions, and metadata for the strains in this study are provided in Supplementary_Table1 (Additional_Supplementary_File1.xlsx). The authors confirm that all supporting data and protocols have been provided within the article or through supplementary data files.

## Introduction

### Oxalotrophy as a multifunctional trait: from soil to gut

Oxalotrophy has been recognized as an ecosystem service owing to its contribution to carbon cycling [2]. Since the isolation of the first oxalate-degrading bacterium from the rumen of sheep grazing on oxalate-rich plants [3], significant advances have highlighted the ecological and physiological importance of oxalotrophy. In *Burkholderia* species, oxalotrophy is exclusively linked to plant growth-promoting strains and is essential for successful plant colonization [4]. The fungal pathogen *Botrytis cinerea* secretes oxalic acid as a pathogenicity factor to facilitate host infection. Interestingly, *Cupriavidus campinensis* degrades this oxalate, providing up to 70% protection against *B. cinerea* infection in *Arabidopsis*, tomato, cucumber and grapevine [5]. Furthermore, oxalotrophic bacteria (OxB) have been proposed as probiotics for treating hyperoxaluria and kidney stones in humans [6]. Despite its multifunctionality, oxalotrophy is not a universal trait among bacteria; its occurrence depends on the presence of specific metabolic pathways and enzymes required for oxalate degradation. To understand the basis of this functional diversity, it is essential to examine the metabolic fate of oxalic acid.

### Oxalate metabolism

Oxalic acid is a ‘Swiss army knife’ among the existing low-molecular-weight organic acids produced by plants, fungi, animals and bacteria. It is also a key component of root exudates, which recruit OxB for plant growth promotion [7]. It plays an important role in various environmental processes, such as soil weathering [8,9], nutrient cycling [10], carbon sequestration through the oxalate–carbonate pathway (OCP) [8], environmental detoxification via metal chelation, bacteria–fungi interaction [11] and land reclamation, with applications spanning environmental sustainability to human health [12,14].

**Fig. 1.**
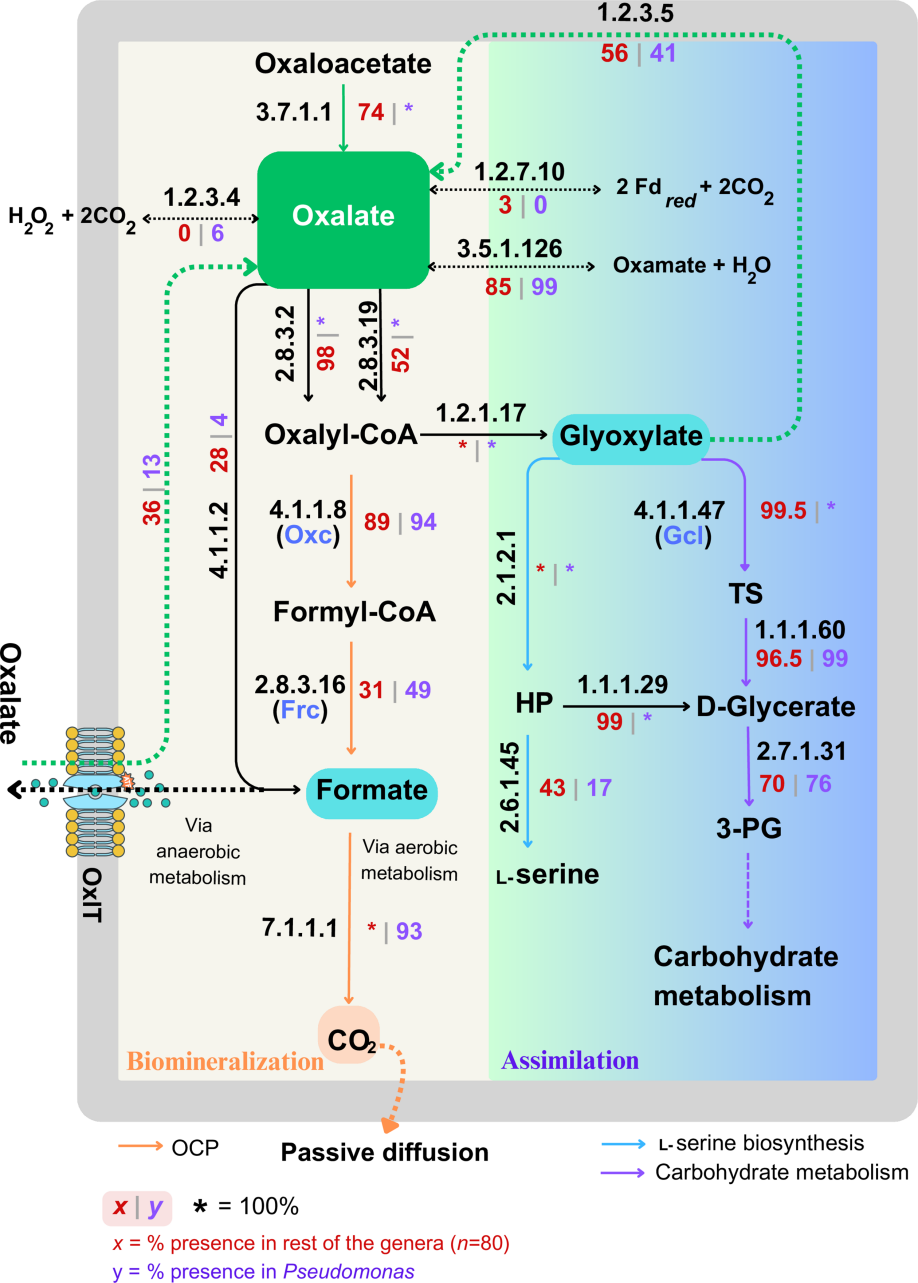
Reactions involved in oxalate metabolism. The pathways shown are in condensed form (i.e. only substrate and main intermediates are shown). Detailed reactions are described by Palmieri *et al*. [11]. The green arrow indicates the biosynthesis route. HP, hydroxypyruvate; PG, phosphoglycerate; TS, tartronate semialdehyde; H_2_O_2_, hydrogen peroxide; Fd_*red*_, Ferredoxin (reduced).

The biogenesis of oxalate is observed in very few bacteria, such as *Pseudomonas fluorescens* ATCC 13525 [15] and *Burkholderia* species [16,17]. Fig. 1 shows the summarized reactions involved in oxalate metabolism. Bacteria can obtain oxalate via three routes. First, as an external carbon source through the activity of an oxalate:formate antiporter under anaerobic metabolism. Second, oxalate can be generated as an intermediate of aerobic energy metabolism via the enzymatic activity of oxaloacetic hydrolase (EC 3.7.1.1), which catalyses the conversion of oxaloacetate into oxalate and acetate. In the third route, oxalate is formed by the breakdown of glyoxylate into oxalate and hydrogen peroxide through the enzymatic activity of glyoxylate oxidase.

The obtained oxalate enters either the OCP or the assimilation pathway. In the OCP route, the oxalate is broken down to formate and carbon dioxide via anaerobic metabolism [12], contributing to inorganic carbon cycling. Carbon dioxide is excreted from the cell by passive diffusion, which is further used to precipitate minerals such as calcium as calcium carbonate [18], thus leading to carbon sequestration. Briefly, oxalate is first converted to oxalyl-CoA by oxalate CoenzymeA (CoA) transferases (EC 2.8.3.2, EC 2.8.3.19). Subsequently, oxalyl-CoA decarboxylase (Oxc; EC 4.1.1.8) catalyses its decarboxylation to formyl-CoA, which is then converted to formate by formyl-CoA transferase (Frc; EC 2.8.3.16). Finally, formate is oxidized to carbon dioxide by Nicotinamide Adenine Dinucleotide (NAD^+^)-dependent formate dehydrogenase (EC 7.1.1.1).

In the assimilation pathway via aerobic metabolism, oxalate is utilized as an intermediate to synthesize amino acids or metabolize carbohydrates through the glycerate pathway. The intermediate oxalyl-CoA is converted to glyoxylate, catalysed by oxalyl-CoA reductase (EC 1.2.1.17). At this metabolic junction, the fate of the oxalate is determined. Under the action of glyoxylate oxidase (EC 1.2.3.5), glyoxylate can be reversibly converted back to oxalate. Alternatively, serine hydroxymethyltransferase (EC 2.1.2.1) and serine–glyoxylate transaminase (EC 2.6.1.45) catalyse its conversion into l-serine, linking oxalate metabolism to amino acid biosynthesis. For carbohydrate metabolism, glyoxylate is channelled into the glycerate pathway. Glyoxylate carboligase (Gcl; EC 4.1.1.47) cleaves glyoxylate to produce tartronic semialdehyde, which is subsequently reduced to d-glycerate by a reductase (EC 1.1.1.60). In the final step, glycerate is phosphorylated to 3-phosphoglycerate by glycerate 3-kinase (EC 2.7.1.31), allowing entry into glycolysis or the Calvin cycle.

In summary, two models have been proposed for oxalate metabolism: the OCP pathway and the assimilation pathway. Key components of the OCP pathway include the enzymes Frc, Oxc and the oxalate:formate antiporter (OxlT). The *frc* and *oxc* genes are commonly organized as operons in many OxB [19]. The assimilation pathway, which operates via aerobic metabolism, involves Gcl and hydroxypyruvate reductase (EC 1.1.1.29) as its key enzymes.

These metabolic routes underscore the versatility of oxalate assimilation in bacteria, connecting oxalotrophy to both energy generation and anabolic processes. Understanding these pathways is especially important in the context of environmental stressors, where oxalotrophy can offer adaptive advantages to microbes, support plant resilience and contribute to broader ecosystem functions.

### Extreme environments and the need for OxB

Arid soils are considered natural environmental stressors for both plants and microbes. They are formed by extremely dry climates and typically have a sandy, coarse texture, resulting in low water retention and low organic matter content. This limits their soil fertility and reduces their ability to support vegetation [20]. Besides, arid soils are often highly calcareous and contain abundant calcium (Ca²^+^), which is challenging for plants to adapt to for survival. Calcicole (chalk-loving) species excel in such environments by tolerating elevated calcium levels and accessing nutrients with low solubility [21]. To survive under these conditions, plants use key strategies, including restricting calcium uptake through apoplastic barriers or sequestering excess calcium as calcium oxalate crystals in specific cell types. This sequestration prevents calcium toxicity and maintains ionic balance, highlighting calcium’s vital role in plant adaptation to calcareous soils [22]. Despite employing these strategies, plants are not always successful, making the challenges of high calcium levels a critical concern for agriculture and future food production [23]. This has prompted interest in biological pathways that mitigate the effects of calcium toxicity, most notably, the OCP pathway of oxalate metabolism, mediated by OxB. Despite the ecological significance of OxB, there is a lack of comprehensive studies investigating the genetic basis of oxalotrophy, particularly in extreme environments such as deserts and mangroves. Therefore, it is necessary to better detect, identify and characterize these micro-organisms to understand their contributions to ecosystem processes and functions.

In this study, we analysed a collection of bacterial genomes, including both previously published sequences [24] and newly assembled genomes from dryland regions and mangroves of the Middle East, to identify bacteria equipped with the enzymatic toolkit required for the assimilation and degradation of oxalate (see Additional File S1, available in the online version of this article). For this purpose, we identified 19 enzymes and one transporter and built a database of high-quality representative enzymes involved in all aspects of oxalate metabolism (Table S1, Additional_Supplementary_File1.xlsx). We focused on and validated oxalotrophy prediction in the family of *Pseudomonads* and revealed the genetic basis for the ability and inability of distinct strains to grow on oxalate.

## Methods

### Oxalate media growth test

All bacteria were tested on Schlegel mineral medium [25] containing calcium oxalate [26]. The first layer contains all components of Schlegel media: Na_2_HPO_4_×12 H_2_O (9.0 g l^−1^), KH_2_PO_4_ (1.5 g l^−1^), NH_4_Cl (1.0 g l^−1^), MgSO_4_×7 H_2_O (0.2 g l^−1^), ammoniacal ferric citrate (0.005 g l^−1^), CaCl_2_ (0.01 g l^−1^), ZnSO_4_×7 H_2_O (50 µg l^−1^), MnCl_2_×4 H_2_O (15 µg l^−1^), boric acid (150 µg l^−1^), CoCl_2_×6 H_2_O (100 µg l^−1^), CuCl_2_ ×2 H_2_O (50 µg l^−1^), NiCl_2_ ×6 H_2_O (10 µg l^−1^), NaMoO_4_ ×2 H_2_O (15 µg l^−1^) and agar (15 g l^−1^). The second layer consists of Schlegel media containing 4 g l^−1^ calcium oxalate monohydrate. The bacteria were grown at 28 °C for 2 days before visual evaluation. As a control, the bacterial strains were grown at 28 °C on Luria-Bertani (LB) agar media (Sigma-Aldrich).

### Genomes and assembly

We sequenced several isolates for this research at the HirtLab, KAUST, and subsequently assembled them in-house with SPAdes v3.15.5 [27]. The assemblies were validated for completeness and contamination with CheckM [28] and for taxonomic assignment using the Average Nucleotide Identity (ANI) [29]. The assemblies thus obtained were annotated using Prokka v1.14.6 [30]. Sequences shorter than 200 bp were filtered out to eliminate noise during annotation. To complete the genomic dataset, we also utilized the published genomes from our repository [24,31,49].

### Identification of enzymes of oxalate metabolism

The enzymes associated with oxalate metabolism were identified in the literature and the BioCyc database [50]. Twenty enzymes were involved in oxalate biosynthesis, assimilation and degradation. We created a local protein sequence database by choosing the best-reviewed, preferably Swiss-Prot sequences [51] belonging to bacteria. When multiple sequences were available, all were included to capture sequence diversity. The accession numbers for the sequences used for the database are shown in Table S1 (Additional_Supplementary_File1.xlsx).

Next, protein sequences obtained from 536 strains were analysed to construct the presence-absence and count number matrix for the identified enzymes. A ‘hit’ was defined by balancing both the sensitivity and specificity [52] of the blast hits [53]. Briefly, the hits were filtered with the following parameters: *E*-value 1E-3, bitscore ≥40, percentage identity ≥30% and at least 60% of query coverage. The similarity clustering based on the whole genomes of 536 bacterial strains was analysed using the Mashtree package v1.4 [54] to generate a genome distance tree, providing a broader context for the distribution of oxalate metabolism enzymes. The tree was visualized with iTOL v6 by mapping each species with its respective traits [55].

### SNP analysis

We performed SNP analysis using Snippy v4.6.0 [56] to investigate the genetic variations between the genomes of interest. The variants were annotated with SnpEff [57] to classify high- and moderate-impact variations. The annotated sequences were further examined for enzyme-related terms (EC numbers) to identify variations potentially affecting enzyme function. Furthermore, the selected protein sequences were aligned using Prank v.170427 [58] to assess sequence-level changes in terms of conservative and non-conservative amino acid substitutions.

## Results and discussion

### Strain isolation and collection

**Fig. 2.**
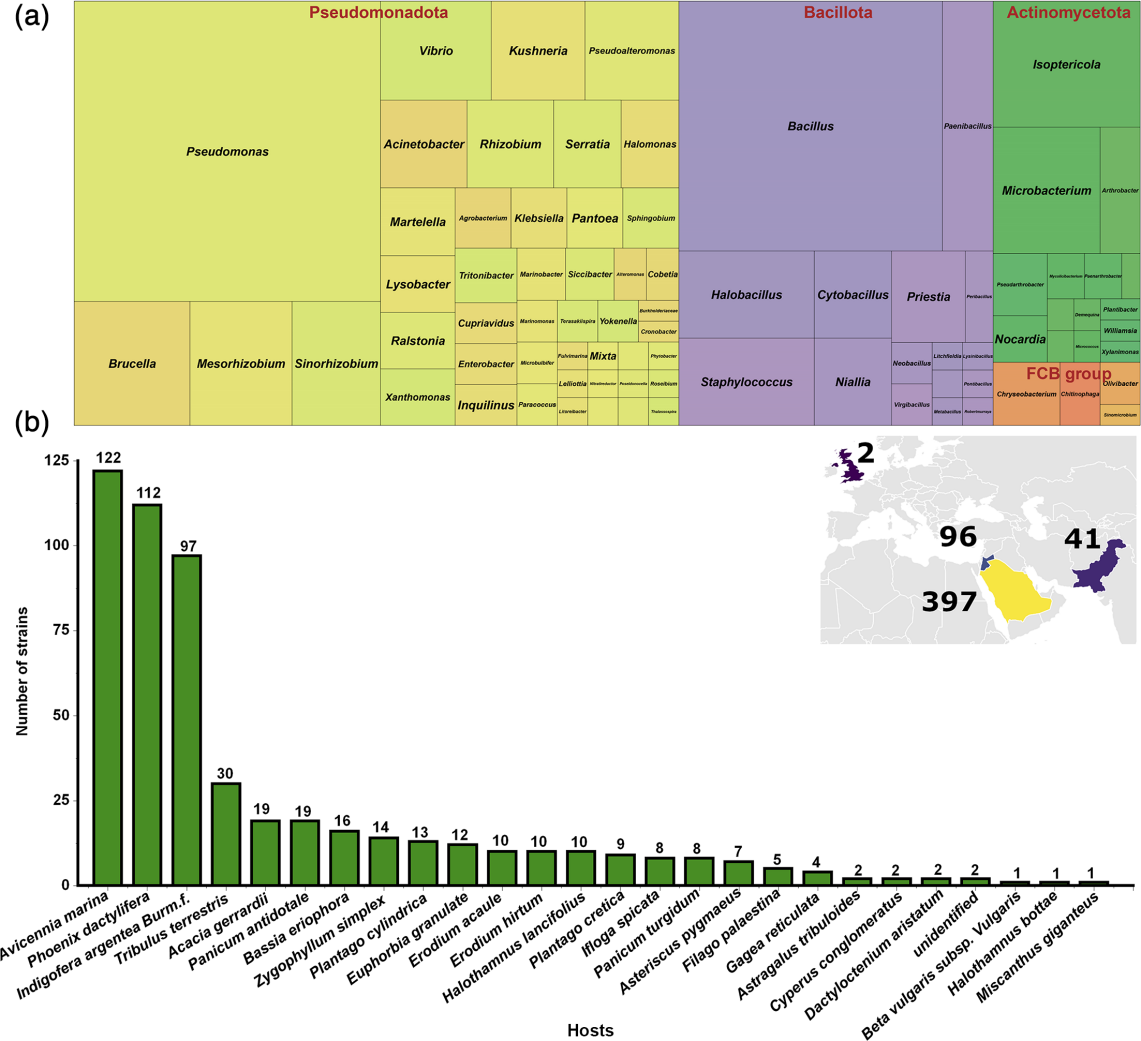
(**a**) Treemap showing the distribution of different genera grouped under the phylum. The box size represents the number of strains identified under each genus. (**b**) Distribution of genera under different hosts. The inset shows the country-wise distribution of the isolated strains.

Our collection included 536 strains from four phyla and 81 genera, isolated from 20 different desert host plants in the Middle East [24]. Among the four phyla (Fig. 2a), Pseudomonadota represents 57% of the isolates, with most of the genus belonging to *Pseudomonas*, and therefore will be highlighted in the analyses. On the other hand, *Bacillota* occupies 20% of the total collection (Fig. 2b). Among the hosts, most strains were isolated from *Avicennia marina* (mangrove biome), followed by the date palm tree, *Phoenix dactylifera*. In the context of the study and the statistics, strains identified as ‘*Pseudomonas*’ will be highlighted in the analyses.

### Genus-wide distribution of enzymes

**Fig. 3.**
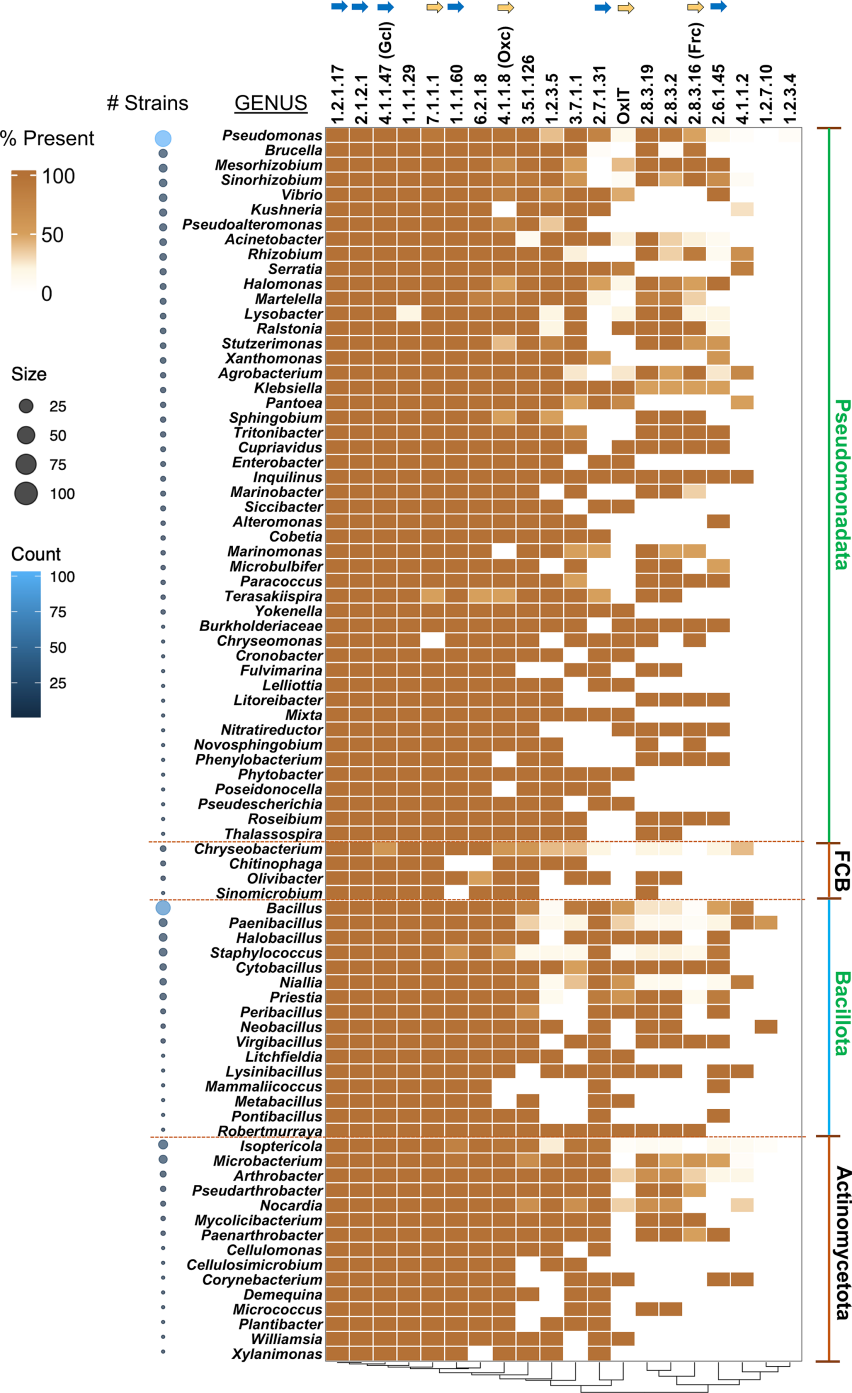
Heatmap showing the genus-wise percentage distribution of the enzymes involved in oxalate metabolism. For each phylum, the genus was sorted in descending order of the number of strains identified (bubble on the left). Blue arrows represent enzymes belonging to assimilation pathways, and the desert-sand-coloured arrows represent the biomineralization pathway enzymes. The bubble colour and the size are proportional to the number of strains in each genus.

To analyse the genus-wide distribution of enzymes, we performed a presence–absence analysis of all the enzymes involved in oxalate metabolism (Fig. 3). The occurrence of each enzyme was calculated as a percentage for each sampled genus, providing an overview of their distribution. As depicted in the heatmap, the highly conserved enzymes are clustered together, whereas the less conserved enzymes are grouped towards the right side. The enzymes involved in the assimilation pathway (Fig. 1) are highly conserved across all genera and participate in anaplerotic reactions, including examples such as 1.2.1.17, 2.8.3.2, 2.1.2.1, 1.1.1.29, Gcl and 1.1.1.60. Conversely, less conserved enzymes like 2.6.1.45, Frc and 4.1.1.20 play crucial roles in the production of key metabolites such as l-serine, formate and oxalate. These less conserved enzymes can, therefore, be characterized as trait specific. Additionally, clustering based on the presence or absence of the enzymes shows three clusters, ranging from ubiquitous presence to rare occurrence (Fig. S1).

The copy number distribution for the enzymes analysed is shown in Fig. S2. The tiles were arranged in descending order of copy number sums, spreading outwards from the circular tree. The first few enzymes near leaf labels show a consistent pattern across all the samples, which could indicate functional specialization or genomic similarity. On the other hand, the pattern is more sporadic towards the outer lanes, suggesting potential metabolic flexibility.

Among the genera studied, the Fibrobacteres-Chlorobi-Bacteroidetes(FCB)group was found to have several holes, even within the highly conserved category. However, this may be attributed to the small sample size of the FCB group, i.e., the total number of strains in the FCB group is 11, compared to 108 *Pseudomonas* and 78 *Bacillus*.

Overall, the percentage distribution of enzymes on the reaction pathways reveals clear preferential routes of oxalate metabolism among the genera. The *Pseudomonas* species prefer to assimilate oxalate via carbohydrate metabolism rather than l-serine biosynthesis. Eighty-three percent of the *Pseudomonas* group exhibit a propensity to produce d-glycerate, either with tartronic semialdehyde or hydroxypyruvate as an intermediate. On the other hand, the rest of the genera exhibit similar preferences for both carbohydrate and amino acid biosynthesis.

### Pathways of oxalotrophy

**Fig. 4.**
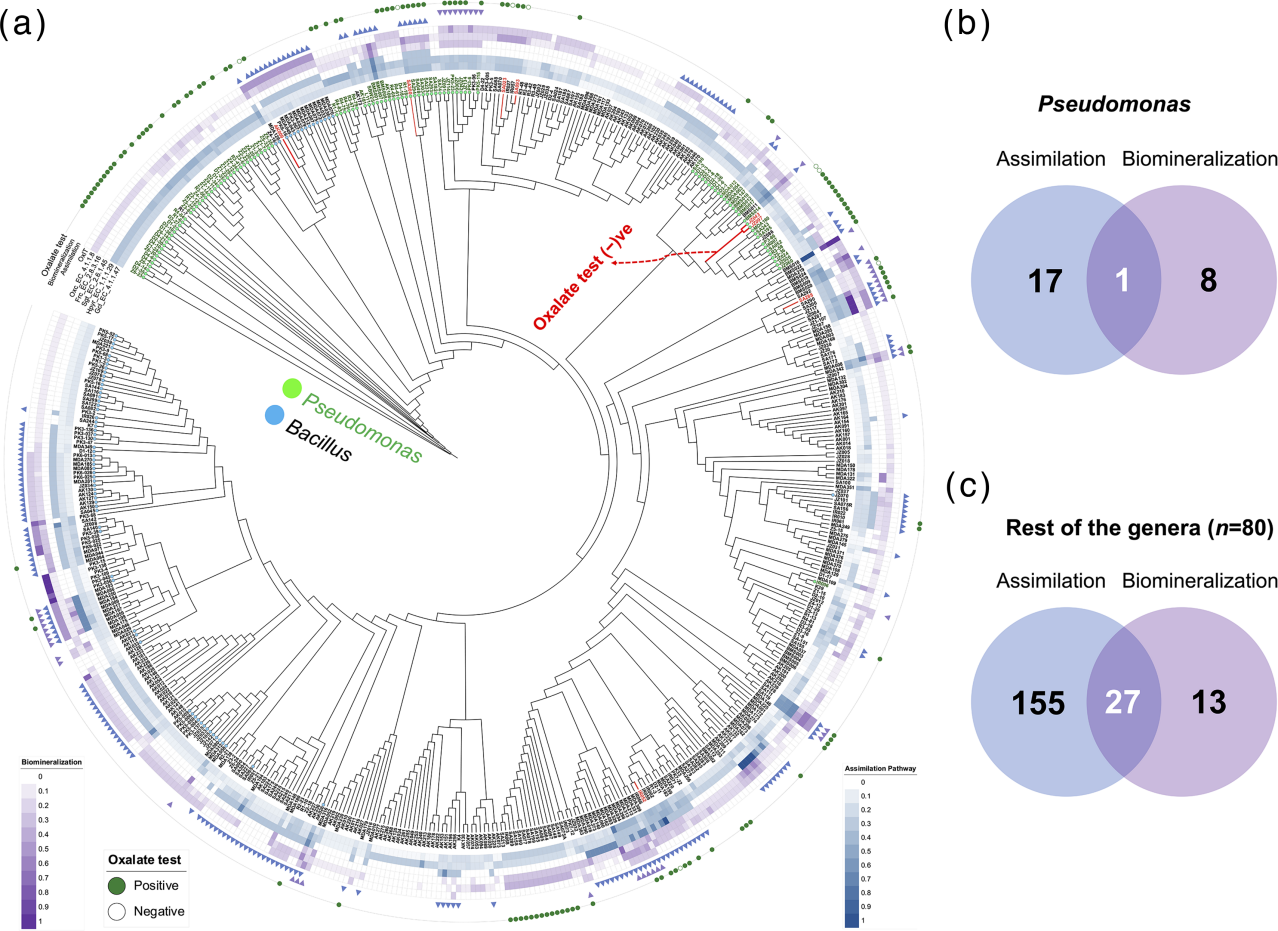
(a) Copy numbers of enzymes in the heatmap for assimilation and biomineralization toolkits. Node edges and labels in green represent the *Pseudomonas* strains, and blue node edges and labels represent *Bacillus* strains. Branches in red represent the oxalate-negative strains. (**b**) Venn diagram of strains in the *Pseudomonas* group that harbour the categorized enzyme repertoire. (**c**) Venn diagram of strains in the rest of the genera. (Strain name: P3-86 *alias* JZ860.)

We categorized the enzymes involved in oxalotrophy into pathway toolkits. The enzymes Oxc (EC 4.1.1.8), Frc (EC 2.8.3.16), and the transporter OxlT were grouped under the biomineralization pathway. Similarly, the enzymes Gcl (EC 4.1.1.47), hydroxypyruvate reductase (EC 1.1.1.29) and serine–glyoxylate transaminase (EC 2.6.1.45) were classified as part of the assimilation pathway. We hypothesized that the completeness of these pathways would confer the ability of oxalotrophy to bacteria. To test our hypothesis, a genomic signature analysis was carried out to determine the presence of the categorized pathways mentioned above. Fig. 4a shows the copy number distribution of enzymes belonging to the categorized pathways.

In our study, nine *Pseudomonas* strains (JZ006, JZ042, JZ091, JZ114, JZ134, MDA012, PK3-6, PK5-115 and PK5-23) were identified with the capacity for biomineralization (Fig. 4b). Interestingly, these strains lacked the enzyme EC 2.6.1.45, which is essential for the l-serine biosynthesis. On the other hand, 18 strains were identified with the complete enzyme set required for oxalate assimilation. Only one strain, *Pseudomonas* sp. MD012*,* isolated from the date palm *P. dactylifera,* contained both the pathways to metabolize oxalate, showcasing its unique metabolic versatility.

In contrast, 40 and 182 strains of other genera contained enzyme sets to achieve biomineralization and assimilation, respectively (Fig. 4c). In theory, 27 strains could convert oxalate into carbon dioxide (CO_2_) and assimilate it as l-serine. Like in *Pseudomonas*, the strains that could perform only biomineralization lacked the key enzyme serine–glyoxylate transaminase EC 2.6.1.45. These findings underscore the critical role of enzyme EC 2.6.1.45 in oxalate assimilation. The strains capable of oxalate assimilation were associated with diverse hosts, including mangrove biomes. As further validation, we tested several strains *in vitro* for their ability to grow on media supplemented with oxalate as the sole organic carbon source.

### Accumulation of SNPs in the key enzyme

**Fig. 5.**
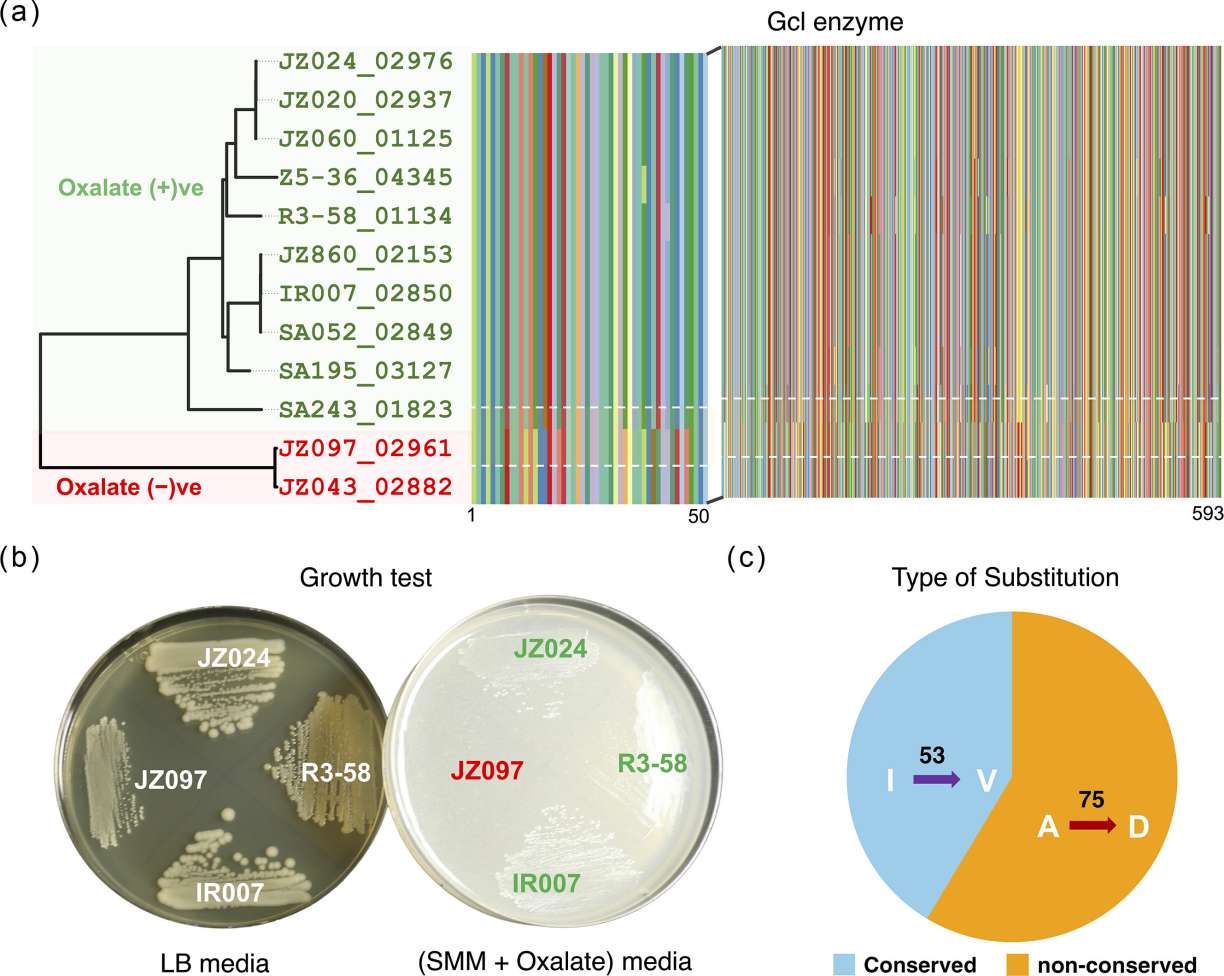
The sub-node of the *Pseudomonas* group tested for oxalate in the growth medium (**a**). The multiple sequence alignment of Gcl amino acid sequences between oxalate-negative and -positive strains. (**b**) Strains tested on Luria-Bertani (LB) media and Schlegel Mineral Media (SMM) supplemented with exogenous oxalate. (**c**) Distribution of conserved and non-conserved substitutions. The amino acid codes indicate: I, Isoleucine; V, Valine; A, Alanine; D, Aspartate.

Among the tested clades, one of the subclades belonging to *Pseudomonas* (Fig. 5a) revealed an interesting pattern, where all the tested strains grew on oxalate media, except for JZ097 and JZ043 (Fig. 5b). To test whether mutations in one or more key enzymes within the oxalate assimilation or biomineralization pathways might be the reason for the observed trait, we performed SNP analysis using *Pseudomonas* spp. JZ024 [36,37], a species with a high-quality and complete genome, as a reference within the subclade. The SNP analyses for all the tested genera are shown in Table S2 (Additional_Supplementary_File1.xlsx). Interestingly, the oxalate-negative strains were found to have missense SNPs in the enzyme Gcl (EC 4.1.1.47). This enzyme is involved in the conversion of glyoxylate to tartronate semialdehyde, which further proceeds to carbohydrate metabolism. The multiple-sequence alignment showed a clear difference in mosaic patterns between the groups (Fig. 5a). In addition, more non-conservative substitutions that affect protein function were observed compared with conserved substitutions (Fig. 5c).

A notable non-conservative and known detrimental substitution observed was alanine to aspartate (A to D). A study published in 1996 investigated the effect of substituting alanine 128 with aspartic acid in the β-subunit of the F-type ATPase (F_o_F_₁_-ATPase) from *Escherichia coli*. This mutation abolished the dimerization of the β-subunit, which is crucial for the proper assembly and function of the ATPase complex [59]. Similarly, the most frequent conservative substitution observed was isoleucine to valine (I to V). Although the mutation is conservative, the position at which it occurs can cause significant disruption in protein structure. In *E. coli,* testing the effects of isoleucine-to-valine and valine-to-isoleucine mutations in thioredoxin resulted in changes in the stability of the protein [60]. Thus, the mutations we observed in Gcl could result in impaired enzyme function, leading to the inability to grow in oxalate media.

The oxalate-carbonate pathway (OCP), facilitated by bacteria, represents a promising mechanism for long-term carbon sequestration in terrestrial ecosystems. This process plays a pivotal role in carbon cycling and contributes to soil carbon storage, offering a natural solution for mitigating atmospheric CO₂ levels. Central to bacterial oxalate metabolism are three key enzymes: Frc, Oxc and the oxalate:formate antiporter (OxlT). These enzymes drive the conversion of oxalate into CO₂, which subsequently precipitates as calcium carbonate, thereby locking carbon in a stable mineral form [12,18]. In our study, we identified several bacterial isolates harbouring these critical enzymes. Two enzymes, oxalate decarboxylase (EC 4.1.1.2) and oxalate oxidase (EC 1.2.3.4), are reported to oxidize oxalate primarily in fungi and lower eukaryotes [2]. Currently, available enzymatic methods for the quantitation of oxalate are based on hydrogen peroxide production via oxalate degradation using EC 1.2.3.4. We identified six *Pseudomonas* species, all isolated from Wadi Rum, Jordan, to harbour these enzymes exclusively. Additionally, four *Pseudomonas* species (BMS010, BMS012, BMS014 and BMS017) from Al Ula, Saudi Arabia, were found to possess EC 4.1.1.2, suggesting distinct metabolic adaptations influenced by the environment or by horizontal gene transfer from other organisms, such as fungi or lower eukaryotes. Incidentally, both these regions belong to the same geographical stretch and share properties of soil and ecology.

The inability of certain bacterial strains to grow on oxalate media, as observed in our results, highlights the intriguing variability in the oxalotrophic potential among different bacteria. Our findings suggest that the accumulation of SNPs in key enzymes might be a reason for the inability of strains to utilize oxalate. The EC 4.1.1.47 protein sequences, coding for Gcl in non-oxalotrophic strains JZ043 and JZ097 [36], were distinct from those of other strains, suggesting potential functional divergence related to oxalate metabolism. These findings underscore significant variability in the oxalotrophic potential among bacterial populations, indicating evolutionary and ecological factors influencing this trait.

As mentioned before, oxalic acid is a key root exudate, shaping rhizosphere microbial communities by recruiting beneficial OxB, such as *Burkholderia*, which promote plant growth and colonization. Oxalotrophy reduces oxalic acid levels, limiting fungal pathogenicity and providing plant protection [4]. Studies show that OxB, such as *Pseudomonas* spp., reduce fungal infections in crops by 30–75% [5,61], highlighting their potential for sustainable agricultural applications. Furthermore, OxB can help mitigate some of the challenges faced in desert agriculture. For instance, their mineral weathering ability can release essential nutrients, such as calcium and magnesium, enriching the nutrient pool in coarse-textured soil. OxB can also break down calcium carbonate into more bioavailable forms, improving root penetration and water infiltration. Furthermore, OxB chelates toxic metals and ions in saline soils, which can reduce soil salinity and alkalinity stress on plants. Due to their broad environmental benefits, our work lays a crucial foundation for harnessing OxB in soil carbon sequestration strategies, especially in arid and semi-arid ecosystems.

## Conclusion

Our study highlights the metabolic versatility of OxB and their critical role in carbon cycling, soil health and plant–microbe interactions. The identification of distinct metabolic pathways, along with genomic variability in key enzymes, underscores the adaptive nature of oxalotrophy. The ability of these bacteria to modulate oxalate levels has significant implications for soil carbon sequestration and sustainable agriculture, particularly in arid environments. Harnessing OxB could offer innovative strategies for enhancing soil resilience and mitigating climate change, warranting further research into their functional stability and ecological impact in natural ecosystems.

## Supplementary material

10.1099/mgen.0.001587 Uncited Supplementary Material 1.

10.1099/mgen.0.001587 Uncited Supplementary Material 2.

## Abbreviations

Ca²^+^: calcium
Frc: formyl-CoA transferase
Gcl: glyoxylate carboligase
OCP: oxalate–carbonate pathway
OxB: oxalotrophic bacteria
Oxc: oxalyl-CoA decarboxylase
