# Supplementary material for: Genomic and functional divergence of oxalate metabolism pathways in bacteria from contrasting ecosystems
Source: Microb Genom. 2026 Feb 20;12(2):001587. doi: 10.1099/mgen.0.001587 (PMC12927642; doi:10.1099/mgen.0.001587)
Supplement: Uncited Supplementary Material 1. [file mgen-12-01587-s001.pdf]

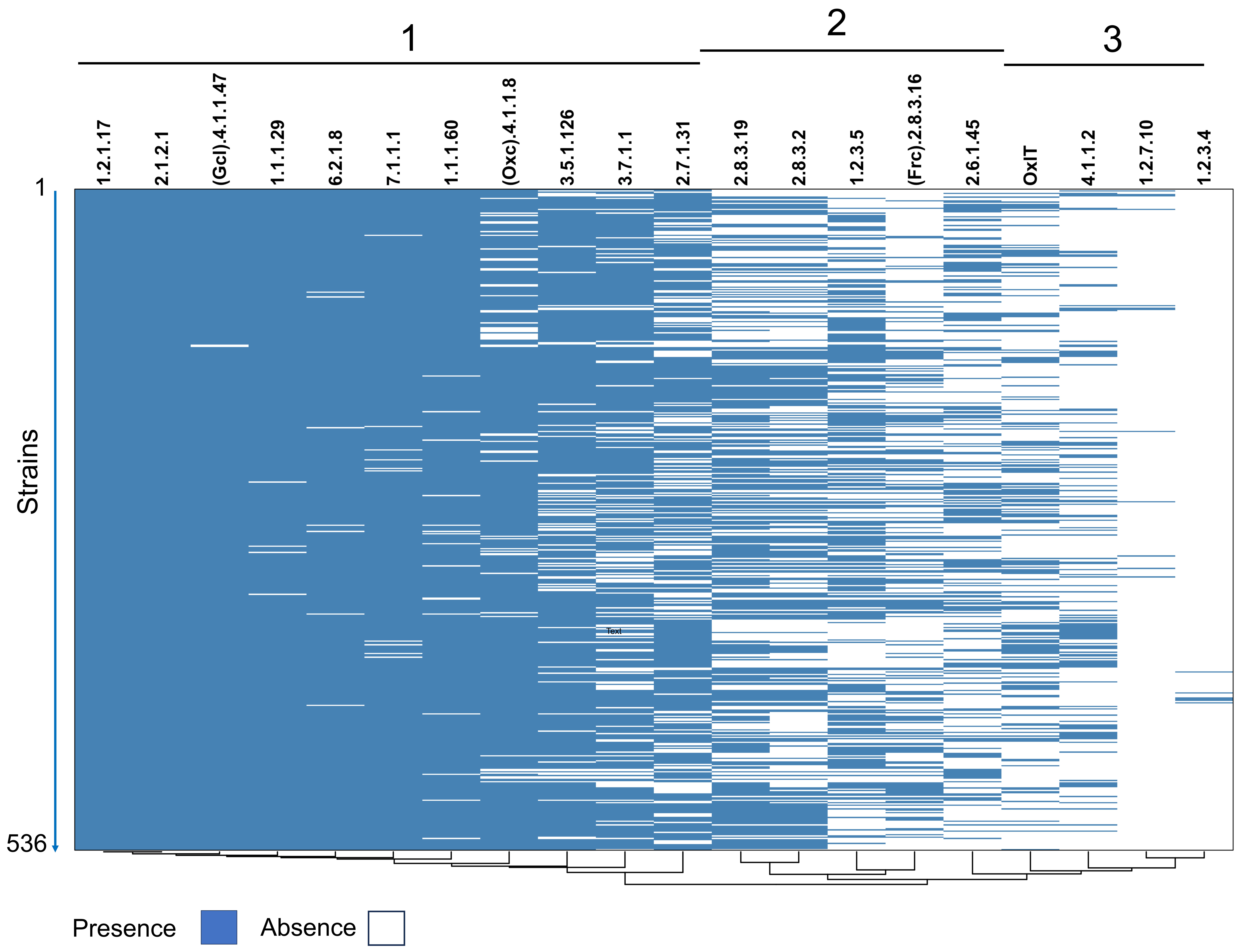
1
2
3
1.2.1.17
2.1.2.1
(Gcl).4.1.1.47
1.1.1.29
6.2.1.8
7.1.1.1
1.1.1.60
(Oxc).4.1.1.8
3.5.1.126
3.7.1.1
2.7.1.31
2.8.3.19
2.8.3.2
1.2.3.5
(Frc).2.8.3.16
2.6.1.45
OxlT
4.1.1.2
1.2.7.10
1.2.3.4
1
536
Strains
Text
Presence
Absence

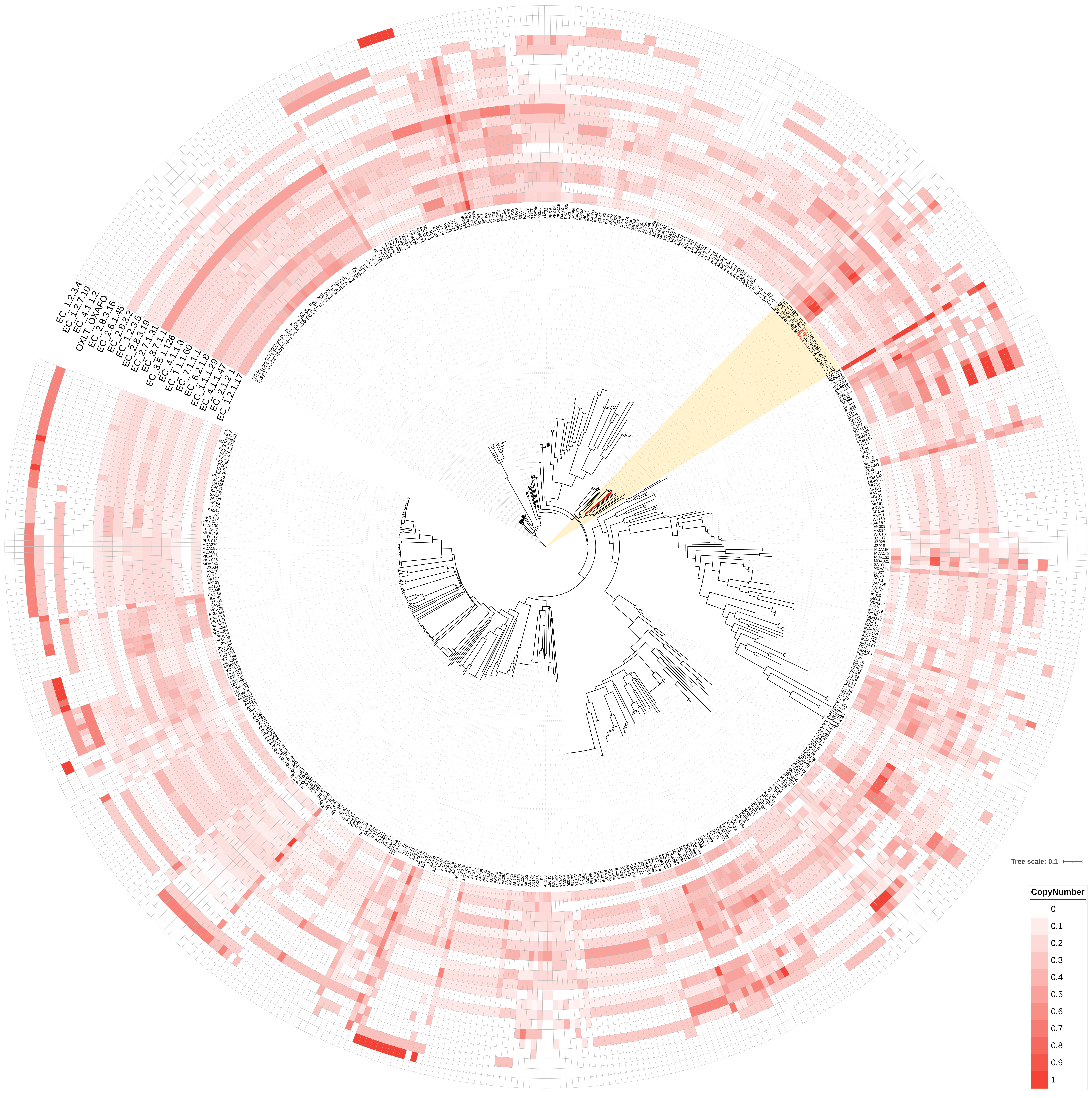
EC_1.2.3.4
EC_1.2.7.10
EC_4.1.1.2
OXLT_OXAFO
EC_2.8.3.16
EC_2.6.1.45
EC_2.8.3.2
EC_1.2.3.5
EC_2.8.3.19
EC_2.7.1.31
EC_3.7.1.1
EC_3.5.1.126
EC_4.1.1.8
EC_1.1.1.60
EC_7.1.1.1
EC_6.2.1.8
EC_1.1.1.29
EC_4.1.1.47
EC_2.1.2.1
EC_1.2.1.17
Tree scale: 0.1
CopyNumber
0
0.1
0.2
0.3
0.4
0.5
0.6
0.7
0.8
0.9
1

SF1: Heatmap of presence or absence of Enzymes across the collection of strains.

SF2: Copy number distribution of key enzymes involved in oxalate metabolism. Copy numbers are normalized for each node for visual representation. The highlighted clade shows the *Pseudomonas* subclade where strains tested negative (red) in oxalate media.
